# A Review of Acute Lymphocytic Leukemia (ALL) in the Pediatric Population: Evaluating Current Trends and Changes in Guidelines in the Past Decade

**DOI:** 10.7759/cureus.49930

**Published:** 2023-12-04

**Authors:** Queen L Ekpa, Prince C Akahara, Alexis M Anderson, Omowunmi O Adekoya, Olamide O Ajayi, Peace O Alabi, Okelue E Okobi, Oluwadamilola Jaiyeola, Medara S Ekanem

**Affiliations:** 1 General Practice, Conestoga College, Kitchener, CAN; 2 Mental Health, Manor Clinic, Edmonton, CAN; 3 Pediatric Medicine, St. George’s University, School of Medicine, St. George’s, GRD; 4 Pediatrics, Sage Medical Clinic, Calgary, CAN; 5 Pediatrics, Medway Maritime Hospital, Kent, GBR; 6 Internal Medicine, Obafemi Awolowo College of Health Sciences, Olabisi Onabanjo University, Sagamu, NGA; 7 Pediatrics, University of Abuja Teaching Hospital, Abuja, NGA; 8 Family Medicine, Larkin Community Hospital Palm Springs Campus, Hialeah, USA; 9 Family Medicine, Medficient Health Systems, Laurel, USA; 10 Family Medicine, Lakeside Medical Center, Belle Glade, USA; 11 Family Medicine, Lagoon Hospitals, Lagos, NGA; 12 General Medicine, Babcock University Teaching Hospital, Ilishan-Remo, NGA

**Keywords:** financial costs, survival rates, resistance, relapse, toxicity, acute leukemia, childhood, challenges, treatment guidelines, cure rates

## Abstract

Acute lymphocytic leukemia (ALL) is a commonly diagnosed cancer in children. Despite technological advancements to improve treatment and survival rates, there has been a steady increase in the incidence of ALL and treatment failures. This paper discusses the pathogenic interaction between genetic and environmental factors leading to childhood ALL. It evaluates the current treatment guidelines and notable obstacles leading to resistance, relapse, and treatment toxicities. The review evaluates a 10-year trend in the management guidelines of pediatric ALL through a systematic literature review of records from 2012 to 2023. Findings show that improvement in the five-year survival rates, notwithstanding rates of relapse and incurable diseases, is still high. Furthermore, several risk factors, including an interplay between genetic and environmental factors, are largely contributory to the outcome of ALL treatments and its overall incidence. Moreover, huge financial costs have remained a significant challenge in outcomes. There remains a need to provide individualized treatment plans, shared decision-making, and goals of care as parts of the management guidelines for the best possible outcomes. We expect that future advancements will increase overall survival rates and disease-free years.

## Introduction and background

Pediatric cancer is any childhood cancer occurring between birth and 14 years of age, generally including those less than 18 years old [1,2]. Acute lymphoblastic leukemia (ALL) is the most common cancer diagnosed in children less than 15 years, with median age of diagnosis at 15 years [2,3]. ALL expresses a bimodal peak, more likely to occur at one to four years of age compared to infants and those aged 10 years and above [3]. ALL is a diverse hematological disease distinguished by its proliferation of immature lymphoid cells in the bone marrow, peripheral blood, and other organs representing almost 80% of all pediatric acute leukemias [3]. Almost 6,000 new cases and 1,500 deaths from ALL were estimated to occur in 2019 at an age-adjusted incidence rate of 1.38 per 100,000 individuals annually in the US, with a peak incidence at one to four years of age [2,3]. There has been a steady increase in the incidence of pediatric ALL since 1975, despite considerable improvements in survival rates over the years [2]. The incidence is higher in children of American Indian, Native Alaskan, and Hispanic descents. However, it is twice as high in White than Black kids [3]. In Canada, 59 children between zero and 14 years were reported to die from ALL between 2016 and 2020 [4]. 

This paper explores the pathogenic interplay of genetic and environmental factors in childhood ALL. It succinctly evaluates the evolving management guidelines in the US and Canada over the last decade, addressing the reasons for these changes. Additionally, the discussion covers current treatment guidelines and obstacles linked to resistance, relapse, and treatment toxicities.

Pathophysiology

ALL is a complex disease involving genes, biology, and environmental factors. Wide-based genomic studies demonstrate a close interplay between inherited and somatic genetic alterations in the biology of ALL [2]. Several cytogenetic studies carried out in the last three decades, have provided information on the pathogenesis of ALL. With the advent of high-throughput genomics and next-generation sequencing (NGS) technologies, knowledge of specific molecular lesions and critical pathways of leukemogenesis has exponentially increased [2]. ALL arises from hematopoietic cells in either the B-cell precursor (BCP-ALL/ B-ALL) or T-cell lineages (T-ALL). Both BCP-ALL and T-ALL immunophenotypes comprise multiple subtypes defined by chromosomal alterations believed to be leukemia-initiating lesions [5]. Common translocations in children with B-ALL include t(12;21) [ETV6-RUNX1] (25%), t(1;19) [TCF3-PBX1] (5%), t(9;11) [BCR-ABL1] (3%) and translocations involving the MLL gene with various partner fusion genes (5%). Gains in whole chromosomes or high-hyperdiploidy (>50 chromosomes) account for 25% of childhood ALL, whereas hypodiploidy (<44 chromosomes) accounts for approximately 1% of cases [6]. However, 20% of newly diagnosed BCP-ALL cases do not belong to any of the known genetic subtypes and, therefore, are given a subtype referred to as B-other [5].

B-ALL

Eighty-five percent of childhood ALL are of the B-lineage, with BCP-ALL being the most common [6,7]. Prevailing chromosomal abnormalities affecting 50% of patients in this subtype of ALL are t(12;21)(p13;q22) representing ETV6-RUNX1 fusion and high hyperdiploidy characterized by gains of whole chromosomes. Other common chromosomal abnormalities are summarized in Table 1.

**Table 1:** Chromosomal rearrangements in BCP-ALL Reference: [7]

Aberration	t(12;21)(p13;q22)	t(9;22)(q34;q11.1)	t(1;19)(q23;p13.3)	t(17;19)(q22;p13)	t(4;11)(q21;q23)
Fusion gene	ETV6-RUNX1	BCR-ABL1	TCF3-PBX1	HLF-TCF3	MLL-AFF1

T-ALL

Structural rearrangements of chromosomes typical for T-ALL contribute to the activation of oncogenes mainly by translocation in TCR loci (Table 2). Regions especially vulnerable to aberration are 14q11 in the locus TCRA/D and 7q34 in TCRB. Regular thymopoiesis is supervised by several transcription factors, but their presence at fragile sites of chromosomes poses a serious threat of mechanism failure [7]. Among the most significant factors, NOTCH1 should be listed as responsible for the self-renewal of hematopoietic stem cells and the development of T cells. Its mutation alters at least five substantial pathways involving TAL1, LYL1, LMO1, LMO2, TLX1, TLX3, and MYC. The pathology is reflected in the increased expression of oncogenes (e.g., MYC) and decreased expression of suppressor genes p16/INK4A, p14/ARF (CDKN2A), TP53, and RB [7]. 

**Table 2:** Chromosomal rearrangements in T-ALL Reference: [7]

Abnormality	del(1)(p32)	t(1;14)(p32;q11)	t(11;14)(p13;q11)	t(8;14)(q24;q11)	inv(7)(p15q34)/t(7;7)(p15;q34)	t(9;22)(q34;q11)	t(5;14)(q35;q11)	t(11;19)(q23;p13.3)	t(10;14)(q24;q11)
Gene	SIL-TAL1	TAL-TCRA/D	LM02-TCRA/D	MYC-TCRA/D	LMO1-TCRA/D	BCR-ABL1	TLX-TCRA/D	MLL-MLLT1(ENL)	bHLH-TCRA

Down Syndrome ALL

Patients with Down syndrome are approximated to have a 20-fold increased risk of developing ALL, although the precise role of the extra chromosome 21 in leukemogenesis is not properly understood [6]. These patients have low frequencies of T-ALL and common ALL translocations, such as ETV6-RUNX1 [6].

Predisposing factors and etiopathogenesis

Childhood leukemia (CL) arises as a result of an interplay between genetic and environmental factors. These risk factors are poorly understood, and those already identified only contribute to 10% of associated risks [5,8]

Genetic Risk Factors

Several genetic factors are implicated:

Inherited genetic susceptibility: Germline mutations are an important risk factor in developing childhood ALL [8]. Studies conducted have identified susceptibility loci in ARID5B, CEBPE, BMI1, CDKN2A/2B and others that are associated with an increased risk of developing childhood ALL [8]. Some of these genes account for racial predilection in certain racial groups. ARID58 is found in Hispanics and could account for the higher incidence rates seen in this group [8,9]. Rare germline mutations in some developing hematopoietic genes, such as ETV6, PAX5, or IKZF1, have also been shown to predispose children to ALL [8]. Rare syndromes, including Cornelia de Lange syndrome and Rubinstein-Taybi syndrome, are also associated with childhood ALL [8]. Other associated syndromes with a high risk of developing childhood ALL include Noonan syndrome, Down syndrome, Fanconi anemia, and constitutional mismatch repair deficiency [8]. There is a 4.4% prevalence of pathogenic germline mutations in known cancer-causing genes in children and adolescents with ALL. With the advancements in technology and the development of novel techniques for identifying cancer predisposition syndromes, new germline risk variants are emerging [8].

Epigenetics: Epigenetic changes, a hallmark of cancer, are characteristic of childhood ALL, with genetic, environmental, and metabolic factors contributing to these changes [8]. Inducing DNA methylation may be a mechanism by which some associated environmental factors predispose children to this condition [8]. Several large-scale epigenome-wide association studies have reported associations of relevant maternal exposures during pregnancy, including tobacco smoking, air pollution, and body mass index, with DNA methylation in offspring neonatal blood. For instance, decreased methylation at aryl-hydrocarbon receptor repressor (AHRR) CpG cg05575921 has been associated with exposure to maternal smoking during pregnancy [8]. Accumulating evidence suggests that the pathogenesis and phenotypic characteristics of leukemic cells are the results of a combination of specific targeted and genome-wide alterations of DNA methylation [10].

Environmental Factors

There are environmental factors associated with ALL that have been documented in the past. Notable ones are as follows:

Infections: The idea that infection plays a causal role in ALL is about 100 years old; two hypotheses have been proposed to explain how infections predispose to ALL, namely, Kinlen's “population mixing” hypothesis and Greaves's “delayed infection” hypothesis [8,9]. Both hypotheses propose that ALL is a consequence of an abnormal response to common infections [8]. Indications for an infectious etiology came from observing leukemia cases occurring in closer spatial and temporal proximity than would be expected if they occurred independently from one another [8]. A study was conducted, which showed strong evidence of clustering of CL at the time of diagnosis for children aged zero to five years, an age range including the peak incidence for leukemia at two to four years [8]. Results were similar for ALL. Such clustering in space and time could be explained by “mini-epidemics” of a single infection leading to local clusters of leukemia cases, which are observed from time to time [8]. The risk for Infections and developing ALL is not limited to the postnatal period, but studies have also found maternal infections to be a potential risk factor [8,9,11]. The most frequently studied infections associated with ALL are viral infections, followed by bacterial infections and fungal infections [8]. Specific ALL-associated Infections include influenza infections, whereas CL in general was associated with influenza, rubella, and varicella infections during pregnancy [8].

Ionizing radiation (IR): High and moderate doses have been recognized for decades as strong risk factors associated with CLs; exposure specifically during childhood has been associated with a higher risk of developing cancer as compared to adulthood exposure [8]. The dose-response for leukemia after IR exposure is described as linear-quadratic, slowly changing at low doses, but rapidly at high doses. Evidence for developing leukemia when exposed to low-dose radiation defined as radiation <100 mGy is sparse, but increased doses are associated with significant risks [8].

Extremely low-frequency magnetic fields: Research is still ongoing on the relationship between extremely low-frequency magnetic fields and the risk for ALL [8]. The presented study results are relatively consistent in that they show a higher risk for developing CL and specifically ALL with magnetic field exposures above 0.3 or 0.4 μT [8].

Other identified risk factors include high and low birth weight and sex, with boys more often affected than girls, pesticide exposure, air pollution, paint, paternal tobacco smoking, prelabour cesarean delivery, and maternal intake of cured meats [8,5,12]. N-nitroso precursors in cured meats when converted to carcinogenic components by stomach acids are then transported through the placenta to the developing fetus [5].

Diagnostic challenges: the role of genomic analysis and molecular markers

ALL is a multifaceted condition characterized by various subtypes [10], which makes its diagnosis and treatment quite challenging. Genomic analysis and molecular markers are pivotal in addressing these diagnostic hurdles and enhancing the treatment of ALL [13]. The utilization of genomic analyses has transformed our comprehension of the molecular classification of ALL, prompting more efforts and results in the clinical care of ALL. This integration aims to enhance precise risk assessment/stratification and, in certain instances, enable targeted therapeutic approaches [14,15]. Here are some key diagnostic challenges and how genomic analysis and molecular markers can help:

Subtyping ALL: ALL is not a singular disease but rather a group of related disorders [10,14]. Determining the precise subtype of ALL holds paramount importance when making treatment choices. Genomic analysis helps to discern distinctive genetic irregularities, including chromosomal translocations and gene mutations, to categorize ALL subtypes [14]. In addition, genomic analysis, notably transcriptome sequencing, has unveiled numerous novel subtypes not apparent through cytogenetic examination due to hidden or varied rearrangements and sequence mutations that serve as pivotal driver factors [14].

Risk stratification: Evaluating the risk level of ALL in a patient is vital for effective treatment planning. Molecular markers serve as valuable tools in assessing a patient's risk by pinpointing high-risk genetic mutations and chromosomal abnormalities linked to a less favorable prognosis. This information assists in determining the appropriate intensity and duration of treatment [14]. Conventional karyotyping has been the traditional method for identifying genetic abnormalities in ALL children, aiding in both diagnosis and risk stratification. However, genomic studies have widened the landscape of ALL diagnosis and risk assessment [15]. These studies have revolutionized our comprehension of ALL's molecular taxonomy and have been instrumental in refining the classification of ALL subtypes. These genetic alterations discovered further hold significant implications for the advancement of innovative and precisely targeted therapeutic approaches [15].

Minimal residual disease (MRD) monitoring: MRD denotes the presence of a small number of cancer cells that might persist in a patient's body following treatment. Advanced genomic analysis methods, such as polymerase chain reaction (PCR) or NGS, identify MRD even at very low levels [16]. This heightened sensitivity facilitates a more precise evaluation of the response to treatment and enables healthcare providers to make necessary adjustments to therapy based on this information [16].

Treatment selection: Targeted therapies are more potent and less harmful than conventional chemotherapy. Genomic analysis can pinpoint precise genetic mutations or rearrangements that are susceptible to drug treatment. For example, tyrosine kinase inhibitors (TKIs) can effectively target Philadelphia chromosome-positive ALL, while immunotherapies that target CD19 have shown promise in treating B-cell ALL [13]. It is important to note that several severe side effects of ALL treatments are relatively rare, and their occurrence varies significantly depending on the specific treatment regimen. Genome-wide approaches have demonstrated substantial promise in revealing new mechanisms and identifying novel risk factors associated with drug toxicities [13].

Predicting treatment response: Certain genetic markers can forecast a patient's reaction to specific treatments. For instance, the presence of particular mutations can indicate resistance to certain drugs, enabling doctors to opt for alternative therapies from the outset [13]. The realms of pharmacokinetics, pharmacodynamics, and pharmacogenomics have unveiled pivotal factors contributing to variations in treatment responses among different patients, which can significantly impact clinical outcomes [13]. Recent advancements in technologies for examining both inherited and acquired genome variations have expedited the identification of genomic factors linked to intrinsic drug resistance [13]. These discoveries also offer potential avenues for addressing drug resistance by targeting proteins that, when overexpressed or functionally enhanced, contribute to resistance against traditional anti-leukemic agents. Continuous exploration of genome-wide variations in both germline and somatic DNA (genetic and epigenetic) promises to provide deeper insights into the mechanisms of drug resistance and yield fresh treatment strategies to further enhance the efficacy of ALL therapy [13].

Relapse prediction: Genomic analysis is instrumental in identifying patients with a heightened risk of relapse based on genetic factors. This can prompt closer monitoring and potentially more aggressive treatment approaches in such cases [14]. The intricate subclonal nature of ALL is now well established, and the evolution of clonal populations during treatment and at the point of relapse has been meticulously investigated through genomic sequencing and single-cell analysis [14]. Furthermore, inherited genomic variations, especially in specific ethnic or racial groups, can also contribute to the risk of relapse due to variations in drug metabolism or the acquisition of unique somatic mutations. Monitoring the dynamics of mutation clearance during the initial induction therapy or watching for the emergence of mutations associated with relapse can help identify patients who could benefit from early modifications to their treatment plans [14].

Research and drug development: The genomic analysis of patients with ALL is a valuable contribution to ongoing research efforts and the development of novel therapies. The discovery of new genetic markers and a deeper understanding of their roles in the disease hold the potential for more effective treatments in the future. Collaborative group studies have played a pivotal role in refining the molecular classification of ALL and have significantly advanced the strategies for personalized treatment by identifying fresh targets for therapeutic intervention. These collaborative research initiatives should persist in addressing numerous unresolved questions and issues [13]. NGS studies, which encompass the analysis of the genome, transcriptome, and epigenome of patients, will provide a comprehensive view of all genetic variations contributing to the development of leukemia and influencing treatment outcomes. However, interpreting the intricate interactions among proteins and pathways will remain a formidable challenge in this pursuit [13]. Genomic analysis and molecular markers play indispensable roles in the diagnosis and treatment of ALL. These tools offer crucial insights for subtype identification, risk assessment, treatment decisions, ongoing monitoring, and research endeavours. Collectively, they contribute to enhancing patient outcomes and advancing the development of targeted therapies for this complex condition.

Justification

Impact of Advancements in Managing ALL

ALL treatments have been associated with increased risks for adverse outcomes, such as late mortality, secondary malignancies, and neurological, cardiac, endocrine, orthopedic, and social/psychological disorders [17,18,19,20]. Meta-analysis of ALL survivors treated without cranial radiation demonstrated significant impairment in IQ and other neurocognitive domains [21]. Incorporating effective immunotherapy into ALL therapy would enable the intensity of conventional chemotherapy to be decreased and thereby reduce associated toxicity, leading to further improvements in the survival and quality of life of patients with ALL [22]. New immunotherapeutic approaches provide a major paradigm shift in oncology overall, often curing previously incurable patients [18]. Treatment intensity based on risk-based stratification is the cornerstone of treatment [23,24]. Targeted therapy has revolutionized the treatment of poor-prognosis pediatric ALL with specific genetic abnormalities [24]. The profiling of the gene expression of ALL is important in determining prognostic factors and choosing an appropriate treatment [15,25,26].

Five-Year Survival Rates in Canada and the US

**Figure 1:**
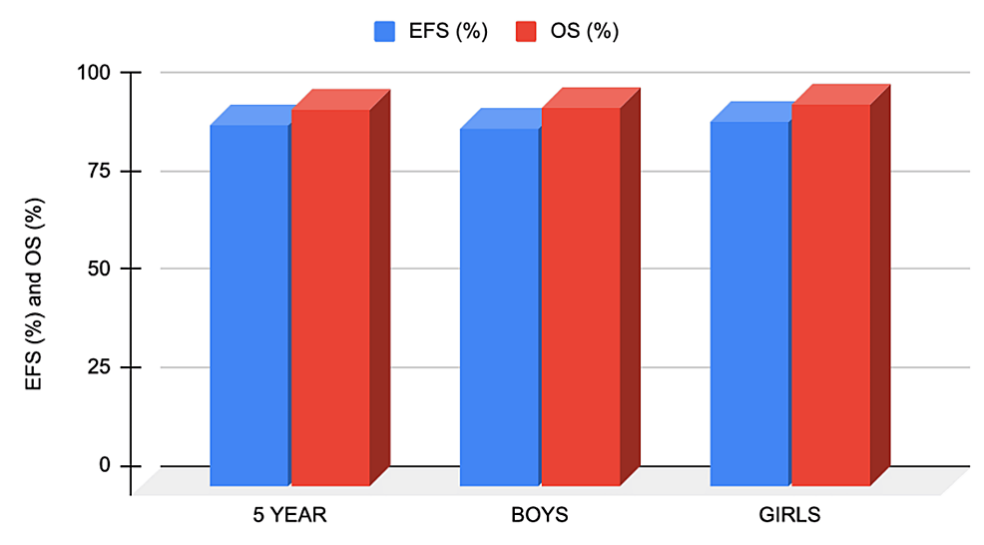
EFS and OFS by sex in B-ALL Comparison of survival rates by age and sex [30]. Note: Disease-free survival (DFS) rates were comparable in both sexes. EFS: event-free survival, OS: overall survival.

**Figure 2:**
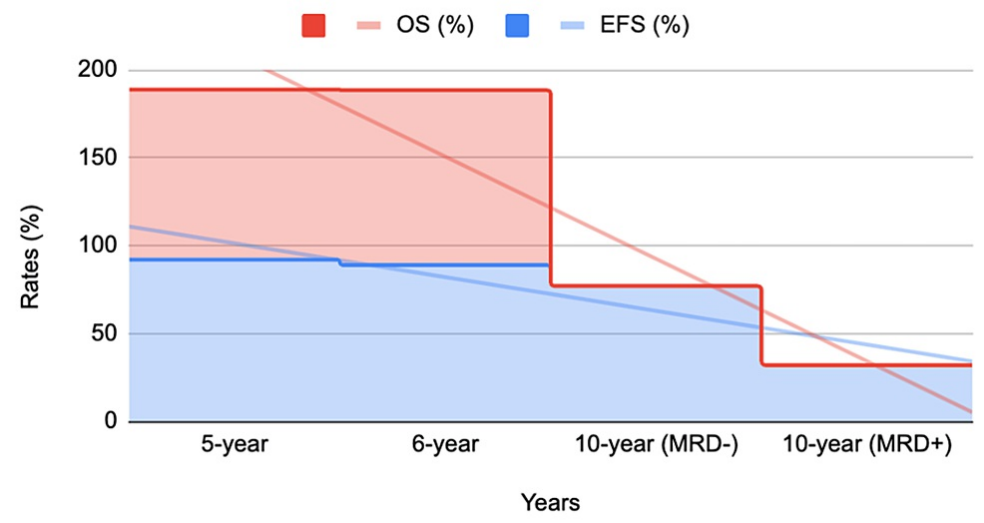
Comparison of survival rates in ALL over five-, six-, and 10-year post-therapy Trends in pediatric ALL survival rates between five and 10 years following treatment [34,35]. EFS: event-free survival, OS: overall survival, MRD: minimal residual disease

The survival rate of children diagnosed with ALL has shown significant improvement over the years with advancing therapeutic regimens. This has in turn impacted the five-year survival rate percentage to over 90% [17,21,27-29] with rates ranging between 78% and 91% [30], as shown in Figure 1. Treatment regimens involving central nervous system (CNS)-directed treatments, such as intrathecal methotrexate (MTX), have proven to help in the improvements seen as evident by these numbers [17,31,32]. Other factors, such as being euglycemic during treatment [33] and the reduction in the use of vincristine (VCR) and dexamethasone pulse treatment, have also been shown to improve the therapeutic outcomes in the pediatric population diagnosed with ALL [31]. Five- to 10-year survival rates following the treatments are illustrated in Figure 2.

Improvement in Technology, Drugs, Diagnosis, Follow-Up Modalities, Insurance Compliance, and Awareness

Over the years, much research and clinical work have improved the diagnosis, management, follow-up modalities, and awareness of ALL in children. MRD testing by flow cytometry compared to no testing improves clinical outcomes in all newly diagnosed patients with precursor B-cell ALL over their lifetime, and it represents good value for money [36]. In present times, immunotherapy is one of the innovative treatment choices against cancer. Owing to their ability to modulate the host immune system while attacking cancer cells, antibody and cellular-based therapies have gained enormous interest from academic researchers and pharmaceutical entrepreneurs in treating various malignant tumors [37].

The curability of childhood ALL was established by pioneer trials conducted in the 1960s and 1970s. By current standards, the early trials used what is now characterized as a non-intensive treatment [38]. Long-term survivors in these initial trials received limited chemotherapy with not more than five essential drugs, including steroids, vinca alkaloids, antimetabolites, and alkylating agents (specifically, prednisone, VCR, 6-mercaptopurine (6-MP), MTX, and cyclophosphamide) [38]. The diagnosis was based on cell morphology, only a few antileukemia agents were available, and there was little knowledge of chemotherapy pharmacokinetics or pharmacodynamics. Since then, long-term survivors have been monitored to guide the successive refinement of therapy [38]. In retrospect, cures induced by the early approaches occurred primarily among cases classified today as lower-risk ALL. Additional drugs were introduced into frontline and maintenance therapy to improve overall outcomes. Table 3 shows the course of ALL drug therapy.

**Table 3:** Trends in ALL drug therapy MTX: methotrexate, 6-MP: 6-mercaptopurine, HSCT: hematopoietic stem cell transplantation, CRT: cranial radiotherapy, CNS: central nervous system, MAB: monoclonal antibody, CAR T: chimeric antigen receptor T-cell, ALL: acute lymphocytic leukemia

Trends in ALL therapy over the years	
Years	Medications
1940s-1950s	Aminopterin, mercaptopurine, and MTX
1960s	6-mercaptopurine and MTX
1970s	6-MP, MTX, HSCT (relapsed), asparaginase, craniospinal irradiation (prophylactic)
1980s	CRT, intrathecal MTX, cytarabine, hydrocortisone
2008	Nelarabine for CNS relapse
In recent years	MABs (blinatumomab and CAR T)

Predictably, the intensification of the therapy worsened toxicity. Efforts to reduce the adverse effects of treatment required improvements in supportive care and multidisciplinary approaches, particularly in the treatment and prevention of infections [38]. The efficacy of increased treatment intensity was tested very carefully but in a trial-and-error fashion, i.e., a more minor versus more significant number of drugs, half versus total chemotherapy dosage, and short versus long duration of therapy were assessed in randomized trials [38]. The treatment has been intensified by using more agents and higher dosages. As systemic chemotherapy improved with increasing knowledge of pharmacokinetics and pharmacodynamics, changes in CNS-directed therapy were possible. CNS irradiation was initially viewed as a requisite of curative treatment. However, its use has decreased substantially and, in some recent protocols, has been eliminated, a significant achievement considering its carcinogenic potential and adverse effect on the developing brain [38].

Nelarabine, a nucleoside analogue, was approved by the US Food and Drug Administration after the publication of two phase-II trials in pediatric and adult patients with relapsed or refractory T-ALL or T-LBL in 2008 and has been successful in remission of relapsed and refractory T-ALL [39,40]. Nelarabine has shown effectiveness in reducing CNS relapses and improving disease-free survival rates in higher-risk ALL, regardless of age or race [40]. Improvements in survival rates for children treated for ALL has led to an increased focus on improving quality of life by mitigating cognitive late effects as children diagnosed with ALL are at increased risk of development of cognitive late effect. This population often exhibits deficits in attention, working memory (WM), and processing speed that emerge one to five years following diagnosis [41].

Cogmed® is a specific computerized intervention designed to improve WM and has demonstrated efficacy in children with neurodevelopmental and acquired attention problems [41]. A 2013 pilot study by Hardy et al. on childhood cancer survivors showed a compliance rate of 85%, indicating adequate feasibility and acceptability of Cogmed when used with this population, as well as overall adherence to, and both participants' and caregivers' general satisfaction with, and efficacy of Cogmed among pediatric cancer survivors [41]. The efficacy of this program with pediatric cancer survivors was examined by Conklin et al. in 2015 where participants who completed training with Cogmed showed statistically significant improvements in WM, attention, processing speed, and declines in executive dysfunction [41].

## Review

Treatment strategies and guidelines

Since 1975, the overall incidence of childhood cancers, including ALL, has been increasing. Nevertheless, from 1975 to 2020, mortality from childhood cancer decreased by roughly 50%. Aggressive advances in treatment guidelines played a huge role in this progress [3]. The American Cancer Society outlines chemotherapy as the main treatment for ALL, with length of treatment typically two to three years [42]. Children with ALL are stratified into risk groups, which allows them to receive the correct types and doses of the given drugs. As expected, the intensity of treatment depends on the risk group. The risk groups are classified as low risk, standard risk, high risk, or very high risk. The higher the risks that patients are determined to have, the more intense the treatment. There are a few prognostic factors when determining which risk group a child belongs to, which include age at diagnosis, initial white blood cell (WBC) count, ALL subtype, sex, number of chromosomes in the leukemia cells (ploidy), chromosome changes (e.g., translocations), and response to initial treatment. The two most important prognostic factors, however, are age at diagnosis and initial WBC count [42,43]. Improvement of treatment for ALL retired formerly used clinical features to categorize patients, as they were proven to no longer be independently associated with treatment outcomes. Some of those formerly used factors include sex, race/ethnicity, presence of a mediastinal mass, organomegaly or lymphadenopathy, and hemoglobin and platelet counts, in addition to serum immunoglobulin levels. The Children Oncology Group Protocol (COG-AALL) is the standard protocol for curing ALL in the US [43].

The treatment is given in three main phases, namely, induction, consolidation (also known as intensification), and maintenance, as summarized in Table 4 [16,44]. The goal of the first phase is to induce complete remission, typically lasting four weeks [3]. The standard induction treatment options in America for newly diagnosed childhood ALL include chemotherapy; the following agents are used with or without an anthracycline (either doxorubicin or daunorubicin): VCR, corticosteroid (prednisone or dexamethasone), asparaginase (pegaspargase, calaspargase pegol, asparaginase *Erwinia chrysanthemi* and native *Escherichia coli* L-asparaginase (unavailable in the US), and intrathecal chemotherapy [2,3,45]. The standard consolidation/intensification and maintenance therapy (post-induction therapy) include chemotherapy. During pre-maintenance therapy for all groups, CNS-directed therapy is provided. Some protocols (COG, St. Jude Children’s Research Hospital) provide ongoing intrathecal chemotherapy during maintenance, while others (Berlin-Frankfurt-Münster (BFM)) do not. The most commonly used intensification schema is the BFM backbone. This therapeutic backbone includes the following: an initial consolidation following the initial induction phase with intrathecal therapy, cyclophosphamide, low-dose cytarabine (Ara-C), and 6-MP. An interim phase exists where intrathecal therapy and four doses of high-dose MTX with leucovorin rescue are given. Delayed intensification, which is similar to agents and schedules used during induction and initial consolidation phases, is employed, and the maintenance phase typically consists of daily oral 6-MP, weekly low-dose MTX, and sometimes VCR and a corticosteroid is administered intermittently, while intrathecal therapy is continued [2,3]. In 2017, the first gene therapy - an autologous chimeric antigen receptor (CAR) T-cell therapy - indicated for children and young adults with relapsed and/or refractory CD4+ ALL was approved in the US. Due to its high levels of toxicities, consensus guidelines were developed for its use and management in treating pediatric patients [46]. 

**Table 4:** Stages of ALL treatment by guidelines VCR: vincristine, 6-MP: 6-mercaptopurine, CMP: cyclophosphamide, ARA-C: cytarabine, MTX: methotrexate, ALL: acute lymphocytic leukemia

Stages of ALL treatment		
	Goal	Drugs used
Induction	Induce complete remission	Anthracyclines, VCR, 6-MP, and intrathecal chemotherapy
Consolidation/intensification	Intensify remission	Initial consolidation: intrathecal therapy, CMP, low-dose ARA-C, and 6-MP
		Interim intensification: intrathecal therapy and four doses of high-dose MTX + leucovorin rescue
		Delayed intensification: same as induction and initial intensfication drugs
Maintenance	Maintain remission	Daily oral 6-MP, weekly low-dose MTX +/- intermittent (VCR and corticosteroid), continued intrathecal therapy

In Canada, the treatment of ALL has undergone several changes in the last decade, although chemotherapy is the mainstay of treatment, which usually lasts two to four years and is given in three to four phases [47]. One of the novel advances includes embracing CAR T-cell therapy as the standard of care for relapsed or refractory B-cell ALL [48]. The one-year survival rate for relapsed or refractory B-ALL is about 30%, but the adoption has been limited by the high cost of accessing the treatment. Thus, it is reserved for patients with B-ALL who are resistant to first-line treatments, relapsed on hematopoietic stem cell transplant (HSCT), or have had two or more relapses [48]. Asparaginase has been modified to different forms and is a backbone in ALL treatments. Polyethylene glycol (PEG)-linked asparaginase called pegaspargase is also a standard of treatment in ALL, and asparaginase *Erwinia *is used for patients allergic to pegylated asparaginase. Pegaspargase is commonly used in the intensive phase of treatment, while 6-MP is used in the maintenance phase [48].

Methods, search strategy, and search criteria

We conducted a systematic literature review of management guidelines in pediatric ALL in the last decade. We searched the PUBMED database electronically for articles from 2012 to 2023 with disease-specific keywords: “acute lymphoblastic leukemia," “pediatric,” “children,” and “treatment,” without any language limitations, which yielded 415 articles. Additional electronic search of the Canadian Medical Association Journal (CMAJ) site (37 results), Canadian Pediatric Society (29 results), Canadian Research Data Centre Network (two results), Leukemia and Lymphoma Society of Canada (one result), Canadian Institute for Health Information (four results), and Canadian Cancer Registry (Statistics Canada: 15 results), for studies specific to Canada with the above disease-specific keywords. Secondary information was obtained from the National Cancer Institute, National Comprehensive Cancer Network, American Cancer Society, Canadian Cancer Society, and UpToDate textbook.

Inclusion and exclusion criteria

**Figure 3:**
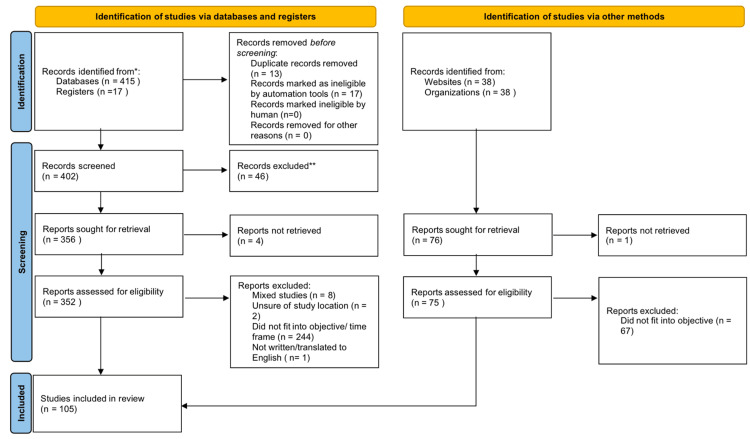
Methodology results Preferred Reporting Items for Systematic Reviews and Meta-Analyses (PRISMA) flow diagram for systematic reviews

Pre-screening eliminated duplicate articles. Studies included in our analysis met the following criteria: (1) papers focused on pediatrics age 18 years and below, (2) papers published between 2013 and 2023, (3) papers written or translated to English, and (4) papers focused on ALL. ​​Excluded studies did not meet these criteria. Figure 3 presents the methodology results using the Preferred Reporting Items for Systematic Reviews and Meta-Analyses (PRISMA) flow diagram for systematic reviews.

Discussion

Challenges and Controversies in the Guidelines

Despite improvement in outcomes and survival without relapse up to 85-95%, there are significant adverse events, relapse to ALL treatment and treatment resistance, with only about half of first relapses surviving long term [43,49,50,51,52]. There is an increased risk of relapse in patients of Black and Hispanic ancestries and lower survival rates in Hispanic kids, as well as in genetic inheritance that interfere with drug clearance and leukemic resistance [53,54]. Relapse typically occurs in the bone marrow and sites, such as the CNS and gonads [55]. Down syndrome patients are at increased risk for cytotoxicities [56]. Intensive cytotoxic drug therapy, site-targeted radiotherapy, and HSCT have been the mainstays of treatment until the advent of immunotherapeutic regimens. Graft versus host disease is a common complication of HSCT [57]. Critical considerations include therapies for relapsed and refractory ALL, complete remission, and the role of immunotherapies [49,50,58]. Early relapse is noted in 6-MP resistance, a mainstay maintenance therapy in many protocols, due to mutations in the NT5C2 and PRPS1 genes and in the SETD2 gene in a smaller number of patients, and loss of the MSH6 gene [43,59]. The expression of the MYC gene, a proto-oncogene, is stimulated by the TCF3::HLF gene, leading to self-renewal and cell proliferation of malignant cells, which is extensively involved in cancer cells' resistance to treatment. The phosphoinositide 3-kinases (PI3K) pathway has also been implicated in cell-mediated adhesion during chemotherapy, leading to relapse and poor prognosis; although PI3K inhibitors can reduce this, they further lead to toxicities [60,61].

Immunotherapy is a landmark treatment for pediatric ALL with poor prognosis, with the hope of improving outcomes while reducing the use of cytotoxic chemotherapy; however, there has been resistance to immune therapies, and post-reinduction treatment with blinatumomab showed little difference in disease-free survival rates, although it has a safer toxicity profile [43,50,58]. Resistance to immune therapies is suspected to be from continued exposure to modified cells, common in patients with relapsed ALL who have a negative expression of CD19 and antigen loss or down-regulation, leading to a known antibody [43]. While patients with relapsed T-ALL have salvage therapies, such as CAR T-cell, which further favors nelarabine use in newly diagnosed T-ALL, there are no such exceptional salvage options for B-ALL [40]. Furthermore, there is no biomarker to determine patients who will effectively respond to CAR T-cell therapy and other immunotherapies, and access to CAR T-cell remains a challenge [48,62]. MicroRNAs have also been notorious for drug resistance and toxicity, even though they avoid cytokine release to reduce cytokine-related adverse effects [63]. Hypereosinophilia is an accompanying complication of ALL supposedly due to increased plasma cytokine release [64]. Some drugs, such as venetoclax, although not a standard therapy, may lead to acquired resistance due to the activation of already-existent cellular pathways of antiapoptosis [43].

MRD is crucial in determining the response to and outcome of ALL treatments [16,65]. MRD is a leukemia cell population that survived chemotherapy, radiotherapy, and immunotherapy treatments or from transformed secondary ALL cells, thus leading to relapse. Other sequelae of post-ALL treatments include cardiomyopathy, secondary leukemias, tumors, and neurophysiological disturbances. Notably, hyperdiploidy in B-ALL is associated with higher survival rates and good prognosis, but up to one-quarter still have relapses and recurrences due to mutations in hyperdiploid cells [43]. Furthermore, gene translocations leading to the TCF3::HLF fusion gene are very resistant to traditional chemotherapy, although they rarely occur [43]. About a quarter of patients with Philadelphia (Ph+) ALL develop resistance to TKIs due to point mutations in BCR::ABL1 genes. Ph-like ALL is also strongly predicted to be resistant to treatment due to its high number of mutations. Optimal T-cell effector and memory functions, identifying BCL2 protein dependence in the TCF3::HLF gene to inhibit cell apoptosis and improve cell survival, and treatment appropriate to the mutation or active signaling pathway will need to be developed by future advances to improve the effectiveness of immune therapies [24,43,50].

Cranial radiotherapy (CRT), although used to decrease the risk of CNS relapse, has no effect on the overall survival and on the prevention of relapse after conventional first-line therapy [66]. There are no accurate means of identifying children at increased risk of relapse and no way to measure CNS leukemia load, although flow cytometry with low sensitivity is currently being used [67,68]. 

Asparaginase and steroids are significant risk factors for venous thromboembolism (VTE), as they interfere with levels of natural anticoagulants leading to suboptimal treatment responses in ALL patients, and there are currently no proven preventive strategies for VTE during ALL treatment [69,70,71]. Antibodies to asparaginase lead to disease relapse [72]. Glucocorticoids have a significant role in ALL treatments, yet no efficient treatment for their long-term side effects, such as bone loss and osteonecrosis in survivors, exists [73,74]. Impaired vitamin D and bone turnover metabolism further escalates the adverse effects on bone and obesity following chemotherapy [75]. Both asparaginase and steroids have been linked to hyperglycemia and insulin resistance in ALL, leading to infections and treatment resistance [33]. There are also no guidelines to screen or manage the hypertriglyceridemia that follows steroid or asparaginase therapy in ALL treatment, which further increases the risk of thrombosis, but dietary modifications and close monitoring have been proposed for anthracycline cardiotoxicity, although echocardiography monitoring is used [76,77]. Asparaginase and 6-MP have also been linked to severe hypoglycemia in ALL treatments, but the mechanism is unclear [52].

**Figure 4:**
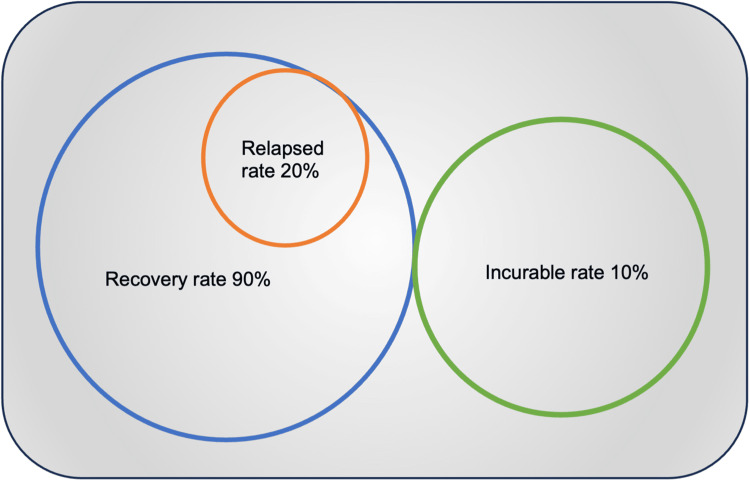
Overview of overall response to B-cell ALL treatment in children Reference: [43]

Depression and anxiety are notably higher in children of Hispanic origin or unhealthy family functioning, especially within the first of diagnosis and treatment, while sleep disturbance and fatigue a common sequelae [20,78,79] These racial and genetic discrepancies pose a significant barrier to ALL therapy [54]. ALL survivors are also noted to develop neurocognitive decline following treatment, affecting about a third of childhood survivors in the US [41,80,81]. It is also challenging to determine adherence or non-adherence to treatment as the maintenance phase is prolonged and consists of oral regimens [82]. Figure 4 below demonstrates a comprehensive response to B-ALL treatment.

Drug Toxicity and Costs

The knowledge of drug toxicities in ALL chemotherapy and immunotherapy is important in therapeutic decision-making. Individual genetic composition has an impact on the pharmacokinetics and drug effectiveness in the body, as well as the toxicity of the drug [6]. The knowledge of drug biology and host genetics is valuable in the management of ALL. In Ontario, Canada, flow cytometry testing for MRD in newly diagnosed precursor B-ALL costs approximately $340,760/year at $1.3 million over three years and $2.4 million over five years [36]. 

Thiopurine S-methyl transferase (TPMT) enzyme deficiency: Excess hematopoietic toxicities and hepatic sinusoidal syndrome have been observed in patients with deficient in TPMT enzyme, leading to the accumulation of active thiopurine metabolites in the cells [6,83].

Blinatumomab: Blinatumomab (a bispecific T-cell engager) toxicities lead to significant neurologic events due to interference of the blood-brain barrier (BBB) by activated T cells, cytokine release syndrome (CRS), infection, and febrile neutropenia [22,43,84]. This co-occurs at the time of T-cell growth and production of proinflammatory cytokines following blinatumomab infusion. While neurologic symptoms of blinatumomab toxicity include malaise, confusion, somnolence, disorientation, encephalopathy, and convulsions, CRS symptoms are pyrexia, nausea, fatigue, and hypotension, whereas headache is a common clinical feature [17,58].

Inotuzumab: Hepatic toxicities, especially sinusoidal obstruction syndrome, were observed to occur with inotuzumab therapy in a randomized trial [13,58]. This is also related to peak levels of inotuzumab.

TKIs

Imatinib: Imatinib majorly causes infections, pancreatitis and osteonecrosis, hepatotoxicity, and gastrointestinal disorders [24].

CAR T-cell: CAR T-cells' adverse effects are observed mainly in the target cells [22,84]. These include CRS, CAR T-cell-related encephalopathy syndrome (CRES), neurotoxicity, febrile neutropenia, prolonged cytopenia, B-cell aplasia, and infections [17, 58,85,86,87]. Neurotoxicity could present as aphasia, delirium, tremor, seizures, and encephalopathy and also as a result of increased permeability of the BBB. The cost of a single infusion of CAR T-cell therapy is about $475,000 in the US, with a charge waiver if treatment fails one month following the infusion. The actual cost of treatment, including supportive care and management of toxicities and complications, such as chronic hypo-gammaglobulinemia, is estimated to reach $1,000,000 [21,37,79].

Asparaginase and pegaspargase: Asparaginase and pegaspargase, *E. coli* derivatives, are essential ingredients in multi-drug therapy regimens for ALL in children and are also associated with known toxicities [58,88,89]. Asparaginase toxicities include clinical hypersensitivity, which is the most common, requiring discontinuation of treatment in up to one-third of patients. Other presentations include subclinical hypersensitivity, silent inactivation of the drug, hypertriglyceridemia, encephalopathy, hepatotoxicity, thrombosis, VTE, myelosuppression, pancreatitis, hyperammonemia (from asparagine breakdown), hypertriglyceridemia, and hyperglycemia [58,69,88,89,90]. It also contributes to osteonecrosis by increasing plasma dexamethasone levels [91].

CMP and 6-MP: These drugs were also observed to contribute to rare cases of immune thrombocytopenic purpura (ITP) in children treated for ALL [92]. This is due to the suspected mechanism of development of ITP in children undergoing ALL treatment following suppression of Treg cells by cyclophosphamide and profound depletion of T cells by 6-MP, leading to immune dysregulation [92].

MTX: This drug is also known to cause neurotoxicities, such as muscle weakness and impaired active range of motion, by an unknown mechanism [58,75]. MTX and its metabolites inhibit 5-aminoimidazole-4-carboxamide ribonucleotide formyl transferase (ATIC), promote adenosine release, and thus modify CNS neuronal responses.

Vincristine: VCR causes peripheral neurotoxicity with a wide array of sensory, motor, and autonomic symptoms [58,75]. Other reported side effects include jaw pain, hoarseness, ptosis, and constipation [58,93].

Nelarabine/arabinosylguanine nucleotide triphosphate (Ara-G): Ara-G, a purine analog used in treating T-ALL unresponsive to or following relapse after at least two chemotherapy regimens, also causes adverse neurotoxic events, such as peripheral motor and sensory neuropathy and seizures [58].

Glucocorticoids (prednisolone, dexamethasone): They are associated with hypertriglyceridemia, decreased bone mineral density, osteonecrosis, and increased bone fractures [73,91,94]. They can also depress the HPA axis, causing impaired stress response and insufficient immune response to infections, and may require hydrocortisone substitution for adrenal hormones [95,96]. They have been implicated as an acute cause of metabolic syndrome and increased cardiovascular risks in ALL survivors [97].

Anthracyclines (doxorubicin, daunorubicin): These have been implicated in cardiotoxicity and apoptosis during ALL treatment [77,98].

CRT: It is associated with a high risk of late neurocognitive sequelae, endocrinopathy, and secondary cancers, as well as depression of hypothalamic and anterior pituitary hormones [99]. Table 5 summarizes common drug toxicities associated with ALL therapy.

**Table 5:** Common ALL drug toxicities a Blinatumomab has little impact on DFS [43]. b CRT has no effect on the overall survival/relapse [43]. CAR T-cell: chimeric antigen receptor T-cell, 6MP: 6-mercaptopurine, CMP: cyclophosphamide, TPMT: thiopurine S-methyl transferase, MTX: methotrexate, VCR: vincristine, Ara-G: arabinosylguanine nucleotide triphosphate, CRT: cranial radiotherapy, CRS: cytokine release syndrome, VTE: venous thromboembolism, ALL: acute lymphocytic leukemia

Common adverse effects of ALL therapy							
Drugs	Imatinib, CAR T-cell, steroids, blinatumomab^a^	Blinatumomab^a^, CAR T-cell	Steroids, asparaginase, pegaspargase	6MP/CMP (TPMT deficiency), inotuzumab, imatinib, asparaginase, pegaspargase	CAR T-cell, MTX, VCR, Ara-G, CRT ^b^	Imatinib, steroids, asparaginase	Imatinib, asparaginase, pegaspargase
Common toxicities	Infections	CRS	VTE, hyperglycemia, hypertriglyceridemia	Hepatotoxicity	Neurotoxicity	Bone disorders/dysmetabolism	Pancreatitis

Adoption by other countries

Globally, different protocols are used to treat childhood ALL; these include the International Collaborative Treatment Protocol for Children and Adolescents with Acute Lymphoblastic Leukemia-AIEOP BFM ALL 2017 in Europe, the Committee for Advanced Therapies (CAT) and the Committee for Medicinal Products for Human Use (CHMP) of the European Medicines Agency, the United Kingdom National Randomized Trial For Children and Young Adults with Acute Lymphoblastic Leukemia and Lymphoma 2011 (UK ALL 2011) in Great Britain, Chinese Children Cancer Group Study-ALL 2015 (CCCG-ALL-2015) in China, and the Japan Association of Childhood Leukemia Study (JACLS) in Japan [43,100]. The common features of these protocols include specific stages of treatment, such as remission, consolidation, and the maintenance of remission based on traditional chemotherapy regimens. Routinely used medications are VCR, daunorubicin (DNR), MTX, doxorubicin (DOXO), ARA-C, CMP, thioguanine (TG), and 6-MP and steroid therapy (prednisone and dexamethasone) at the core of treatment [43].

Asparaginase and CAR T-cells are also used in Europe to treat ALL in all ages [89,100]. A FORUM trial in Germany and France revealed that total body irradiation and etoposide significantly improved survival rates and reduced the risk of relapse [101]. In England, following the successful trial and FDA approval of CAR T-cell (tisagenlecleucel), it was adopted for ALL treatment, and eligibility and prompt access were provided in 10 centers across the nation [102]. Some drugs, such as dasatinib and imatinib used in China, are associated with fatal infections, fungal infections, and pancreatitis, leading to treatment failures in some patients [103].

In France, in the wake of the COVID-19 pandemic, general recommendations include discontinuing or postponing treatment based on the severity of ALL, while involving multidisciplinary care with the Infectious diseases team [104]. Children with ALL were pre-tested for COVID-19 or carefully examined for symptoms if testing was unavailable before treatment was commenced due to the increased risk of severe COVID-19 infections after commencing chemotherapy. Positive test results necessitated a delay in induction therapy, and if patients became positive before induction, repeated testing is done until negative before commencing the intensive phase [104].

In low-income countries, such as in Asia and other developing countries, where diagnostic methods, as well as financial, skill, and logistical resources, pose a huge challenge, recommendations were created using a four-tiered model that considered these factors and created protocols for basic, limited, enhanced, and maximal resources, as well as communication mechanisms to sustain pediatric oncology partnerships [38,105]. All drugs in different phases of this model are given on specific days over a specific period and incorporate the WHO list of essential medications [105].

Limitations

We had challenges gathering Canadian data on epidemiology, treatment guidelines, and specific drug toxicities and costs. A search of Statistics Canada/Canadian Cancer Registry, Canadian Institute of Health Information, Canadian Pediatric Society, and Canadian Cancer Society databases did not provide specific information on pediatric ALL treatment guidelines in Canada. As a result, our paper largely gathered information on the US pediatric population in the last 10 years compared to minimal Canadian information on the same population. We also did not find a unifying international guideline from the WHO in pediatric ALL treatment.

Recommendations

There is a need for more advanced research into individualized treatment plans to improve survival and minimize complications. More research also needs to be done to improve the management of drug toxicity and treatment-related adverse events in the short and long terms. Subsidized treatment costs would also contribute to improved outcomes both in advanced and resource-poor nations.

## Conclusions

The diagnosis, treatment, and overall management of ALL have significantly evolved in the past decade. Drug combination and treatment modalities have largely remained the same. Despite these evolutionary advancements with improvement in disease-free and overall survival rates up to 90%, chances of survival from ALL are not yet optimal. There is no international guideline for pediatric ALL treatment. Treatment relapses and resistance, as well as residual disease, drug toxicities, high cost of newer treatments, and individual genetic variations, have continued to be challenges faced by practitioners in managing this childhood cancer. Treatments for drug toxicity and adverse events from treatment are largely supportive. Financial costs are a huge embargo in resource-poor countries and individuals lacking health insurance. The future is bright with ongoing research to reduce relapse and toxicities with newer treatments. However, emphasis is laid on individualized treatment plans, shared decision-making, and goals of care for improved outcomes. Patients newly diagnosed today have better chances of overall survival, and survivors have a higher probability of disease-free years. We surmise that future advancements will increase the number of disease-free years and overall survival rates beyond the current five-year standard with fewer complications.

## References

[1] (2023). National Cancer Institute: pediatric cancer. https://www.cancer.gov/publications/dictionaries/cancer-terms/def/pediatric-cancer.

[2] Brown P, Inaba H, Annesley C (2012). Pediatric acute lymphoblastic leukemia, version 2.2020, NCCN Clinical Practice Guidelines in Oncology. J Natl Compr Canc Netw.

[3] Brown P, Inaba H, Annesley C (2012). National Cancer Institute. Childhood Acute Lymphoblastic Leukemia Treatment (PDQ®)-Health Professional Version. JNCCN.

[4] (2023). Childhood leukemia statistics. Canadian Cancer Society. https://cancer.ca/en/cancer-information/cancer-types/leukemia-childhood/statistics#:~:text=The%20most%20recent%20incidence%20statistics,diagnosed%20with%20acute%20myelogenous%20leukemia.

[5] Blanco-Lopez J, Iguacel I, Pisanu S (2023). Role of maternal diet in the risk of childhood acute leukemia: a systematic review and meta-analysis. Int J Environ Res Public Health.

[6] Bhojwani D, Yang JJ, Pui CH (2015). Biology of childhood acute lymphoblastic leukemia. Pediatr Clin North Am.

[7] Kęsy J, Januszkiewicz-Lewandowska D (2015). Genes and childhood leukemia. Postepy Hig Med Dosw (Online).

[8] Schmidt JA, Hornhardt S, Erdmann F, Sánchez-García I, Fischer U, Schüz J, Ziegelberger G (2021). Risk factors for childhood leukemia: radiation and beyond. Front Public Health.

[9] Greaves M (2018). A causal mechanism for childhood acute lymphoblastic leukaemia. Nat Rev Cancer.

[10] Nordlund J, Syvänen AC (2018). Epigenetics in pediatric acute lymphoblastic leukemia. Semin Cancer Biol.

[11] Hwee J, Tait C, Sung L, Kwong JC, Sutradhar R, Pole JD (2018). A systematic review and meta-analysis of the association between childhood infections and the risk of childhood acute lymphoblastic leukaemia. Br J Cancer.

[12] Cao Y, Lu J, Lu J (2020). Paternal smoking before conception and during pregnancy is associated with an increased risk of childhood acute lymphoblastic leukemia: a systematic review and meta-analysis of 17 case-control studies. J Pediatr Hematol Oncol.

[13] Pui CH, Yang JJ, Hunger SP (2015). Childhood acute lymphoblastic leukemia: progress through collaboration. J Clin Oncol.

[14] Inaba H, Mullighan CG (2020). Pediatric acute lymphoblastic leukemia. Haematologica.

[15] Xu H, Yu H, Jin R, Wu X, Chen H (2021). Genetic and epigenetic targeting therapy for pediatric acute lymphoblastic leukemia. Cells.

[16] Kruse A, Abdel-Azim N, Kim HN (2020). Minimal residual disease detection in acute lymphoblastic leukemia. Int J Mol Sci.

[17] Kızılocak H, Okcu F (2019). Late effects of therapy in childhood acute lymphoblastic leukemia survivors. Turk J Haematol.

[18] Winters A, Gore L (2019). Moving immunotherapy into the front line in ALL. Hematology Am Soc Hematol Educ Program.

[19] Dupuis LL, Lu X, Mitchell HR (2016). Anxiety, pain, and nausea during the treatment of standard-risk childhood acute lymphoblastic leukemia: A prospective, longitudinal study from the Children's Oncology Group. Cancer.

[20] Myers RM, Balsamo L, Lu X (2014). A prospective study of anxiety, depression, and behavioral changes in the first year after a diagnosis of childhood acute lymphoblastic leukemia: a report from the Children's Oncology Group. Cancer.

[21] Iyer NS, Balsamo LM, Bracken MB, Kadan-Lottick NS (2015). Chemotherapy-only treatment effects on long-term neurocognitive functioning in childhood ALL survivors: a review and meta-analysis. Blood.

[22] Inaba H, Pui CH (2019). Immunotherapy in pediatric acute lymphoblastic leukemia. Cancer Metastasis Rev.

[23] Cooper SL, Brown PA (2015). Treatment of pediatric acute lymphoblastic leukemia. Pediatr Clin North Am.

[24] Lejman M, Kuśmierczuk K, Bednarz K, Ostapińska K, Zawitkowska J (2021). Targeted therapy in the treatment of pediatric acute lymphoblastic leukemia-therapy and toxicity mechanisms. Int J Mol Sci.

[25] Lato MW, Przysucha A, Grosman S, Zawitkowska J, Lejman M (2021). The new therapeutic strategies in pediatric T-cell acute lymphoblastic leukemia. Int J Mol Sci.

[26] Teachey DT, Pui CH (2019). Comparative features and outcomes between paediatric T-cell and B-cell acute lymphoblastic leukaemia. Lancet Oncol.

[27] Raetz EA, Teachey DT (2016). T-cell acute lymphoblastic leukemia. Hematology Am Soc Hematol Educ Program.

[28] Simioni C, Martelli AM, Zauli G, Melloni E, Neri LM (2019). Targeting mTOR in acute lymphoblastic leukemia. Cells.

[29] Andrés-Jensen L, Attarbaschi A, Bardi E (2021). Severe toxicity free survival: physician-derived definitions of unacceptable long-term toxicities following acute lymphocytic leukaemia. Lancet Haematol.

[30] Hucks G, Rheingold SR (2019). The journey to CAR T cell therapy: the pediatric and young adult experience with relapsed or refractory B-ALL. Blood Cancer J.

[31] Angiolillo AL, Schore RJ, Kairalla JA (2021). Excellent outcomes with reduced frequency of vincristine and dexamethasone pulses in standard-risk B-lymphoblastic leukemia: results from Children's Oncology Group AALL0932. J Clin Oncol.

[32] Salzer WL, Burke MJ, Devidas M (2020). Impact of intrathecal triple therapy versus intrathecal methotrexate on disease-free survival for high-risk B-lymphoblastic leukemia: Children's Oncology Group Study AALL1131. J Clin Oncol.

[33] Zhang BH, Wang J, Xue HM, Chen C (2014). Impact of chemotherapy-related hyperglycemia on prognosis of child acute lymphocytic leukemia. Asian Pac J Cancer Prev.

[34] Berry DA, Zhou S, Higley H (2017). Association of minimal residual disease with clinical outcome in pediatric and adult acute lymphoblastic leukemia: a meta-analysis. JAMA Oncol.

[35] Maloney KW, Devidas M, Wang C (2020). Outcome in children with standard-risk B-cell acute lymphoblastic leukemia: results of Children's Oncology Group Trial AALL0331. J Clin Oncol.

[36] (2016). Minimal residual disease evaluation in childhood acute lymphoblastic leukemia: an economic analysis. Ont Health Technol Assess Ser.

[37] Li W, Zhang Y, Kankala RK, Zou L, Chen Z (2022). Antibody and cellular-based therapies for pediatric acute lymphoblastic leukemia: mechanisms and prospects. Pharmacology.

[38] Rivera GK, Ribeiro RC (2014). Improving treatment of children with acute lymphoblastic leukemia in developing countries through technology sharing, collaboration and partnerships. Expert Rev Hematol.

[39] Burkhardt B, Mueller S, Khanam T, Perkins SL (2016). Current status and future directions of T-lymphoblastic lymphoma in children and adolescents. Br J Haematol.

[40] Dunsmore KP, Winter SS, Devidas M (2020). Children's Oncology Group AALL0434: a phase III randomized clinical trial testing nelarabine in newly diagnosed T-cell acute lymphoblastic leukemia. J Clin Oncol.

[41] Fournier-Goodnight AS, Ashford JM, Clark KN (2019). Disseminability of computerized cognitive training: performance across coaches. Appl Neuropsychol Child.

[42] (2023). American Cancer Society: treatment of children with acute lymphocytic leukemia (ALL). https://www.cancer.org/cancer/types/leukemia-in-children/treating/children-with-all.html.

[43] Jędraszek K, Malczewska M, Parysek-Wójcik K, Lejman M (2022). Resistance mechanisms in pediatric B-cell acute lymphoblastic leukemia. Int J Mol Sci.

[44] Horton TM, McNeer JL (2023). Treatment of Acute Lymphoblastic Leukemia/Lymphoma in Children and Adolescents. UpToDate.

[45] Domenech C, Suciu S, De Moerloose B (2014). Dexamethasone (6 mg/m2/day) and prednisolone (60 mg/m2/day) were equally effective as induction therapy for childhood acute lymphoblastic leukemia in the EORTC CLG 58951 randomized trial. Haematologica.

[46] Mahadeo KM, Khazal SJ, Abdel-Azim H (2019). Management guidelines for paediatric patients receiving chimeric antigen receptor T cell therapy. Nat Rev Clin Oncol.

[47] Treatments for childhood ALL (Canadian Cancer Society. Accessed July 22, 2023). Treatments for childhood ALL. https://cancer.ca/en/cancer-information/cancer-types/leukemia-childhood/treatment/acute-lymphocytic-leukemia-all#ci_treatments_for_childhood_all_24_3027_00.

[48] Brown K, Seftel MD, Hay KA (2021). Innovations in cancer immunotherapy: chimeric antigen receptor T-cell therapy (CAR-T). CMAJ.

[49] Hunger SP, Raetz EA (2020). How I treat relapsed acute lymphoblastic leukemia in the pediatric population. Blood.

[50] Witkowski MT, Lasry A, Carroll WL, Aifantis I (2019). Immune-based therapies in acute leukemia. Trends Cancer.

[51] Bhatla T, Jones CL, Meyer JA, Vitanza NA, Raetz EA, Carroll WL (2014). The biology of relapsed acute lymphoblastic leukemia: opportunities for therapeutic interventions. J Pediatr Hematol Oncol.

[52] Jiang M, Ahmet A, Somerville S (2023). Hypoglycemia during treatment of acute lymphoblastic leukemia. Canadian Pediatric Surveillance Program Protocol.

[53] Karol SE, Larsen E, Cheng C (2017). Genetics of ancestry-specific risk for relapse in acute lymphoblastic leukemia. Leukemia.

[54] Lim JY, Bhatia S, Robison LL, Yang JJ (2014). Genomics of racial and ethnic disparities in childhood acute lymphoblastic leukemia. Cancer.

[55] Gaudichon J, Jakobczyk H, Debaize L, Cousin E, Galibert MD, Troadec MB, Gandemer V (2019). Mechanisms of extramedullary relapse in acute lymphoblastic leukemia: Reconciling biological concepts and clinical issues. Blood Rev.

[56] Salzer WL, Burke MJ, Devidas M (2018). Toxicity associated with intensive postinduction therapy incorporating clofarabine in the very high-risk stratum of patients with newly diagnosed high-risk B-lymphoblastic leukemia: a report from the Children's Oncology Group study AALL1131. Cancer.

[57] Owattanapanich W, Leelakanok N, Sanpakit K, Buaboonnam J (2022). A comparison of the clinical outcomes of haploidentical transplantation and other graft sources in acute lymphoblastic leukemia: a systematic review and meta-analysis. Clin Lymphoma Myeloma Leuk.

[58] Śliwa-Tytko P, Kaczmarska A, Lejman M, Zawitkowska J (2022). Neurotoxicity associated with treatment of acute lymphoblastic leukemia chemotherapy and immunotherapy. Int J Mol Sci.

[59] Dieck CL, Ferrando A (2019). Genetics and mechanisms of NT5C2-driven chemotherapy resistance in relapsed ALL. Blood.

[60] Kim HN, Ogana H, Sanchez V, Nichols C, Kim YM (2022). PI3K Targeting in Non-solid Cancer. Curr Top Microbiol Immunol.

[61] Kośmider K, Karska K, Kozakiewicz A, Lejman M, Zawitkowska J (2022). Overcoming steroid resistance in pediatric acute lymphoblastic leukemia-the state-of-the-art knowledge and future prospects. Int J Mol Sci.

[62] Wedekind MF, Denton NL, Chen CY, Cripe TP (2018). Pediatric Cancer Immunotherapy: Opportunities and Challenges. Paediatr Drugs.

[63] Sbirkov Y, Vergov B, Mehterov N, Sarafian V (2022). miRNAs in lymphocytic leukaemias-the mirror of drug resistance. Int J Mol Sci.

[64] Ferruzzi V, Santi E, Gurdo G, Arcioni F, Caniglia M, Esposito S (2018). Acute lymphoblastic leukemia with hypereosinophilia in a child: case report and literature review. Int J Environ Res Public Health.

[65] O'Connor D, Enshaei A, Bartram J (2018). Genotype-specific minimal residual disease interpretation improves stratification in pediatric acute lymphoblastic leukemia. J Clin Oncol.

[66] Vora A, Andreano A, Pui CH (2016). Influence of cranial radiotherapy on outcome in children with acute lymphoblastic leukemia treated with contemporary therapy. J Clin Oncol.

[67] Thastrup M, Duguid A, Mirian C, Schmiegelow K, Halsey C (2022). Central nervous system involvement in childhood acute lymphoblastic leukemia: challenges and solutions. Leukemia.

[68] Thastrup M, Marquart HV, Schmiegelow K (2022). Flow cytometric detection of malignant blasts in cerebrospinal fluid: a biomarker of central nervous system involvement in childhood acute lymphoblastic leukemia. Biomolecules.

[69] Klaassen IL, Lauw MN, van de Wetering MD (2017). TropicALL study: thromboprophylaxis in children treated for acute lymphoblastic leukemia with low-molecular-weight heparin: a multicenter randomized controlled trial. BMC Pediatr.

[70] Liu J, Yang C, Zhang Z, Li Y (2021). Cerebral venous sinus thrombosis in a young child with acute lymphoblastic leukemia: a case report and literature review. J Int Med Res.

[71] Tuckuviene R, Ranta S, Albertsen BK (2016). Prospective study of thromboembolism in 1038 children with acute lymphoblastic leukemia: a Nordic Society of Pediatric Hematology and Oncology (NOPHO) study. J Thromb Haemost.

[72] Chen SH (2015). Asparaginase therapy in pediatric acute lymphoblastic leukemia: a focus on the mode of drug resistance. Pediatr Neonatol.

[73] Velentza L, Zaman F, Sävendahl L (2021). Bone health in glucocorticoid-treated childhood acute lymphoblastic leukemia. Crit Rev Oncol Hematol.

[74] Te Winkel ML, Pieters R, Wind EJ, Bessems JH, van den Heuvel-Eibrink MM (2014). Management and treatment of osteonecrosis in children and adolescents with acute lymphoblastic leukemia. Haematologica.

[75] Ness KK, Kaste SC, Zhu L (2015). Skeletal, neuromuscular and fitness impairments among children with newly diagnosed acute lymphoblastic leukemia. Leuk Lymphoma.

[76] Bhojwani D, Darbandi R, Pei D (2014). Severe hypertriglyceridaemia during therapy for childhood acute lymphoblastic leukaemia. Eur J Cancer.

[77] Lazăr DR, Farcaş AD, Blag C (2021). Cardiotoxicity: a major setback in childhood leukemia treatment. Dis Markers.

[78] Zupanec S, Jones H, McRae L, Papaconstantinou E, Weston J, Stremler R (2017). A sleep hygiene and relaxation intervention for children with acute lymphoblastic leukemia: a pilot randomized controlled trial. Cancer Nurs.

[79] van Hulst AM, Peersmann SH, van den Akker EL, Schoonmade LJ, van den Heuvel-Eibrink MM, Grootenhuis MA, van Litsenburg RR (2021). Risk factors for steroid-induced adverse psychological reactions and sleep problems in pediatric acute lymphoblastic leukemia: a systematic review. Psychooncology.

[80] Zhou C, Zhuang Y, Lin X, Michelson AD, Zhang A (2020). Changes in neurocognitive function and central nervous system structure in childhood acute lymphoblastic leukaemia survivors after treatment: a meta-analysis. Br J Haematol.

[81] Castellino SM, Ullrich NJ, Whelen MJ, Lange BJ (2014). Developing interventions for cancer-related cognitive dysfunction in childhood cancer survivors. J Natl Cancer Inst.

[82] Zeng XL, Heneghan MB, Badawy SM (2023). Adherence to oral chemotherapy in acute lymphoblastic leukemia during maintenance therapy in children, adolescents, and young adults: a systematic review. Curr Oncol.

[83] Stanulla M, Schaeffeler E, Möricke A (2021). Hepatic sinusoidal obstruction syndrome and short-term application of 6-thioguanine in pediatric acute lymphoblastic leukemia. Leukemia.

[84] Brown PA, Ji L, Xu X (2021). Effect of postreinduction therapy consolidation with blinatumomab vs chemotherapy on disease-free survival in children, adolescents, and young adults with first relapse of B-cell acute lymphoblastic leukemia: a randomized clinical trial. JAMA.

[85] Shah NN, Fry TJ (2019). Mechanisms of resistance to CAR T cell therapy. Nat Rev Clin Oncol.

[86] Kotch C, Barrett D, Teachey DT (2019). Tocilizumab for the treatment of chimeric antigen receptor T cell-induced cytokine release syndrome. Expert Rev Clin Immunol.

[87] Frey NV (2019). Chimeric antigen receptor T cells for acute lymphoblastic leukemia. Am J Hematol.

[88] Hijiya N, van der Sluis IM (2016). Asparaginase-associated toxicity in children with acute lymphoblastic leukemia. Leuk Lymphoma.

[89] Heo YA, Syed YY, Keam SJ (2019). Pegaspargase: a review in acute lymphoblastic leukaemia. Drugs.

[90] Burke MJ (2014). How to manage asparaginase hypersensitivity in acute lymphoblastic leukemia. Future Oncol.

[91] Kunstreich M, Kummer S, Laws HJ, Borkhardt A, Kuhlen M (2016). Osteonecrosis in children with acute lymphoblastic leukemia. Haematologica.

[92] Bayhan T, Ünal Ş, Gümrük F, Çetin M (2015). Immune thrombocytopenic purpura during maintenance phase of acute lymphoblastic leukemia: a rare coexistence requiring a high degree of suspicion, a case report and review of the literature. Turk J Haematol.

[93] Tay SY, Foster J, Heczey A, Sitton M (2021). Pediatric oncology patients with vincristine-induced recurrent laryngeal nerve palsy: two case reports and a brief review of literature. Ear Nose Throat J.

[94] Park HW, Tse S, Yang W (2017). A genetic factor associated with low final bone mineral density in children after a long-term glucocorticoids treatment. Pharmacogenomics J.

[95] Rensen N, Gemke RJ, van Dalen EC, Rotteveel J, Kaspers GJ (2017). Hypothalamic-pituitary-adrenal (HPA) axis suppression after treatment with glucocorticoid therapy for childhood acute lymphoblastic leukaemia. Cochrane Database Syst Rev.

[96] Loimijoki T, Lapatto R, Taskinen M (2020). Adrenal function after induction therapy for acute lymphoblastic leukemia in children short: adrenal function in ALL. Eur J Pediatr.

[97] Warris LT, van den Akker EL, Bierings MB (2016). Acute activation of metabolic syndrome components in pediatric acute lymphoblastic leukemia patients treated with dexamethasone. PLoS One.

[98] Pavlovic S, Kotur N, Stankovic B, Zukic B, Gasic V, Dokmanovic L (2019). Pharmacogenomic and pharmacotranscriptomic profiling of childhood acute lymphoblastic leukemia: paving the way to personalized treatment. Genes (Basel).

[99] Follin C, Erfurth EM (2016). Long-term effect of cranial radiotherapy on pituitary-hypothalamus area in childhood acute lymphoblastic leukemia survivors. Curr Treat Options Oncol.

[100] Ali S, Kjeken R, Niederlaender C (2020). The European Medicines Agency Review of Kymriah (tisagenlecleucel) for the treatment of acute lymphoblastic leukemia and diffuse large B-cell lymphoma. Oncologist.

[101] Peters C, Dalle JH, Locatelli F (2021). Total body irradiation or chemotherapy conditioning in childhood ALL: a multinational, randomized, noninferiority phase III study. J Clin Oncol.

[102] Rogosic S, Ghorashian S (2020). CAR-T cell therapy in paediatric acute lymphoblastic leukaemia - past, present and future. Br J Haematol.

[103] Shen S, Chen X, Cai J (2020). Effect of dasatinib vs imatinib in the treatment of pediatric philadelphia chromosome-positive acute lymphoblastic leukemia: a randomized clinical trial. JAMA Oncol.

[104] Rouger-Gaudichon J, Bertrand Y, Boissel N (2021). COVID19 and acute lymphoblastic leukemias of children and adolescents: Updated recommendations (Version 2) of the Leukemia Committee of the French Society for the fight against Cancers and leukemias in children and adolescents (SFCE). Bull Cancer.

[105] Yeoh AE, Tan D, Li CK, Hori H, Tse E, Pui CH (2013). Management of adult and paediatric acute lymphoblastic leukaemia in Asia: resource-stratified guidelines from the Asian Oncology Summit 2013. Lancet Oncol.

